# Supplementary material for: Prognosis of amyotrophic lateral sclerosis with cognitive and behavioural changes based on a sixty-month longitudinal follow-up
Source: PLoS One. 2021 Aug 11;16(8):e0253279. doi: 10.1371/journal.pone.0253279 (PMC8357115; doi:10.1371/journal.pone.0253279)
Supplement: S1 Checklist — (DOCX) [file pone.0253279.s001.docx]

STROBE Statement—checklist of items that should be included in reports of observational studies

|  | Item No. | Recommendation | Page  No. | Relevant text from manuscript |
| --- | --- | --- | --- | --- |
| **Title and abstract** | 1 | (*a*) In abstract:” investigate the clinical prognostic characteristics of ALS patients with cognitive behavioural changes through long-term follow-up in a cohort” | 2 | investigate the clinical prognostic characteristics of ALS patients with cognitive behavioural changes through long-term follow-up in a cohort |
|  |  | (*b*) abstract contains what was done and what was found | 2 |  |
| Introduction | | | |  |
| Background/rationale | 2 | Approximately 50% of amyotrophic lateral sclerosis (ALS) patients have cognitive and behavioural dysfunction in varying degrees and forms. Previous studies have shown that cognitive and behavioural changes may indicate a poor prognosis, and cognitive function gradually deteriorates over the course of disease, but the results of different studies have been inconsistent. In addition, there are relatively limited long-term follow-up studies tracking death as an end point. | 3 | Approximately 50% of amyotrophic lateral sclerosis (ALS) patients have cognitive and behavioural dysfunction in varying degrees and forms. Previous studies have shown that cognitive and behavioural changes may indicate a poor prognosis, and cognitive function gradually deteriorates over the course of disease, but the results of different studies have been inconsistent. In addition, there are relatively limited long-term follow-up studies tracking death as an end point. |
| Objectives | 3 | The purpose of the study was to investigate the clinical prognostic characteristics of ALS patients with cognitive behavioural changes through long-term follow-up in a cohort | 2 |  |
| Methods | | | |  |
| Study design | 4 | Investigate the clinical prognostic characteristics of ALS patients with cognitive behavioural changes through long-term follow-up in a cohort | 3 |  |
| Setting | 5 | A total of 87 ALS patients who met the revised El Escorial diagnostic criteria ^14^ from 2014 to 2015 in Peking University Third Hospital were selected. All patients were followed up for 5 years. The main end point was clinical death. | 3 | A total of 87 ALS patients who met the revised El Escorial diagnostic criteria 14 from 2014 to 2015 in Peking University Third Hospital were selected. All patients were followed up for 5 years. The main end point was clinical death. |
| Participants | 6 | (*a*) The exclusion criteria included the following: (1) other central nervous system diseases, such as Parkinson's syndrome, cerebrovascular disease, brain trauma, or epilepsy; (2) illiteracy; (3) severe physical dysfunction, rendering the subject completely unable to cooperate with neuropsychological examination. According to the diagnostic criteria for ALS-FTSD ^2^, all patients were divided into a pure ALS group, an ALS with bvFTD (ALS-FTD) group, and an ALS with cognitive and behavioural changes group that did not meet the diagnostic criteria for bvFTD. | 3 | (a) The exclusion criteria included the following: (1) other central nervous system diseases, such as Parkinson's syndrome, cerebrovascular disease, brain trauma, or epilepsy; (2) illiteracy; (3) severe physical dysfunction, rendering the subject completely unable to cooperate with neuropsychological examination. According to the diagnostic criteria for ALS-FTSD 2, all patients were divided into a pure ALS group, an ALS with bvFTD (ALS-FTD) group, and an ALS with cognitive and behavioural changes group that did not meet the diagnostic criteria for bvFTD. |
|  |  | (*b*) don’t need |  |  |
| Variables | 7 | Gender, age, education, duration of illness, onset of disease, diagnostic level, FRS-R, ECAS score, FRS-R progression rate from onset to enrolment, FRS-R progression rate from enrolment to death, Survival time | 4 | Table1 |
| Data sources/ measurement | 8* | In the manuscript |  |  |
| Bias | 9 | Mentioned in limitation part | 9 |  |
| Study size | 10 | According to previous study |  |  |

Continued on next page

| Quantitative variables | 11 | The clinical information and neuropsychological Edinburgh Cognitive and Behavioural Amyotrophic Lateral Sclerosis Screen (ECAS) scores of 84 of these ALS patients have been published previously. The clinical information of the other three ALS-FTD patients enrolled during the same period is shown in Table 1 | 3 | The clinical information and neuropsychological Edinburgh Cognitive and Behavioural Amyotrophic Lateral Sclerosis Screen (ECAS) scores of 84 of these ALS patients have been published previously. The clinical information of the other three ALS-FTD patients enrolled during the same period is shown in Table 1 |
| --- | --- | --- | --- | --- |
| Statistical methods | 12 | (*a*) There were three subgroups in this study. When we compared age, years of education, duration of illness, total ECAS score, Functional Rating Scale–Revised (FRS-R) score at enrolment, FRS-R progression rate from onset to enrolment, FRS-R progression rate from enrolment to death, and survival time between each pair of subgroups, a t-test was used for those who met a normal distribution, and the nonparametric Mann-Whitney U test was used for those who did not. The chi-square test was used to compare the distributions of gender, disease onset and diagnostic level between the two groups. When we analysed the correlation between the ECAS score and FRS-R score, FRS-R progression rate from onset to enrolment, FRS-R progression rate from enrolment to death, and survival time, the Pearson method was used when the two groups of variables were linearly correlated; otherwise, the Spearman method was used. A threshold of p<0.05 was used to define significant differences. Statistical analysis was performed using SPSS 18.0 statistical software. | 4 | There were three subgroups in this study. When we compared age, years of education, duration of illness, total ECAS score, Functional Rating Scale–Revised (FRS-R) score at enrolment, FRS-R progression rate from onset to enrolment, FRS-R progression rate from enrolment to death, and survival time between each pair of subgroups, a t-test was used for those who met a normal distribution, and the nonparametric Mann-Whitney U test was used for those who did not. The chi-square test was used to compare the distributions of gender, disease onset and diagnostic level between the two groups. When we analysed the correlation between the ECAS score and FRS-R score, FRS-R progression rate from onset to enrolment, FRS-R progression rate from enrolment to death, and survival time, the Pearson method was used when the two groups of variables were linearly correlated; otherwise, the Spearman method was used. A threshold of p<0.05 was used to define significant differences. Statistical analysis was performed using SPSS 18.0 statistical software. |
|  |  | (*b*) don’t related |  |  |
|  |  | (*c*) use figure | 4 |  |
|  |  | (*d*) use figure | 4 |  |
|  |  | (*e*) don’t related |  |  |
| Results | | | | |
| Participants | 13* | (a) The numbers of patients who completed the follow-up were as follows: 18 patients in the pure ALS group, 21 patients in the ALS with cognitive and behavioural changes group, and 3 patients in the ALS-FTD group. | 4 | The numbers of patients who completed the follow-up were as follows: 18 patients in the pure ALS group, 21 patients in the ALS with cognitive and behavioural changes group, and 3 patients in the ALS-FTD group. |
|  |  | (b) The main reason for the loss to follow-up was that the family members refused to continue follow-up or could not be contacted | 9 |  |
|  |  | (c) use of a flow diagram | 4 | Table1 |
| Descriptive data | 14* | (a) in the table | 5 | Table2 |
|  |  | (b) 18 patients in the pure ALS group, 21 patients in the ALS with cognitive and behavioural changes group, and 3 patients in the ALS-FTD group. | 4 | 18 patients in the pure ALS group, 21 patients in the ALS with cognitive and behavioural changes group, and 3 patients in the ALS-FTD group. |
|  |  | (c) *5 years*  follow-up time | 4 | 5 years follow-up time |
| Outcome data | 15* | *Table 2* | *6* | *Table2* |
| Main results | 16 | (*a*) The survival time of the ALS-FTD group was significantly shorter than that of the pure ALS group (t = 5.33, p < 0.001) or the ALS with cognitive and behaviour changes group (t = 4.25, p < 0.001). The progression rate of ALS Functional Rating Scale–Revised (FRS-R) scores from recruitment to death was significantly faster in the ALS-FTD group than in the ALS group (z = 2.68, p = 0.01) or the ALS with cognitive and behavioural changes group (z = 2.75, p = 0.01). There was no significant difference in survival time (t = 0.52, P = 0.60) or FRS-R score progression rate (z = 0.31, p = 0.76) between the ALS group and the ALS with cognitive and behavioural changes group. The total Edinburgh Cognitive and Behavioural Amyotrophic Lateral Sclerosis Screen (ECAS) score was positively correlated with survival time (r = 0.38, p = 0.01). | 6-7 | Figure2-4 |
|  |  | (*b*) Not applicable |  |  |
|  |  | (*c*) Not applicable |  |  |

Continued on next page

| Other analyses | 17 | Not applicable |  |  |
| --- | --- | --- | --- | --- |
| Discussion | | | | |
| Key results | 18 | The survival time of ALS-FTD patients was shorter than that of pure ALS patients or ALS patients with cognitive and behavioural dysfunction. The total ECAS score may be correlated with survival time. | 9 | The survival time of ALS-FTD patients was shorter than that of pure ALS patients or ALS patients with cognitive and behavioural dysfunction. The total ECAS score may be correlated with survival time. |
| Limitations | 19 | One of the important limitations of this study was that the sample size for follow-up was small; for this reason, multivariate analysis could not be conducted, although such analysis would have been useful for the comparison of survival time, FRS-R progression rate, and the correlation between total ECAS score and survival time. In addition, cognitive and behavioural assessments were not conducted in the middle of the follow-up | 9 | One of the important limitations of this study was that the sample size for follow-up was small; for this reason, multivariate analysis could not be conducted, although such analysis would have been useful for the comparison of survival time, FRS-R progression rate, and the correlation between total ECAS score and survival time. In addition, cognitive and behavioural assessments were not conducted in the middle of the follow-up |
| Interpretation | 20 | Not applicable |  |  |
| Generalisability | 21 | Not applicable |  |  |
| Other information | |  | | |
| Funding | 22 | This work was partially supported by grants from National Natural Science Foundation of China (Project No. 82001350), Peking University Third Hospital Key Clinical Projects (Project No. BYSY2018048), Peking University Third Hospital Cohort Construction Project (Project No. BYSYDL2019002) |  |  |

*Give information separately for cases and controls in case-control studies and, if applicable, for exposed and unexposed groups in cohort and cross-sectional studies.

**Note:** An Explanation and Elaboration article discusses each checklist item and gives methodological background and published examples of transparent reporting. The STROBE checklist is best used in conjunction with this article (freely available on the Web sites of PLoS Medicine at http://www.plosmedicine.org/, Annals of Internal Medicine at http://www.annals.org/, and Epidemiology at http://www.epidem.com/). Information on the STROBE Initiative is available at www.strobe-statement.org.
