# Supplementary figures and images for: Prognosis of amyotrophic lateral sclerosis with cognitive and behavioural changes based on a sixty-month longitudinal follow-up
Source: PLoS One. 2021 Aug 11;16(8):e0253279. doi: 10.1371/journal.pone.0253279 (PMC8357115; doi:10.1371/journal.pone.0253279)

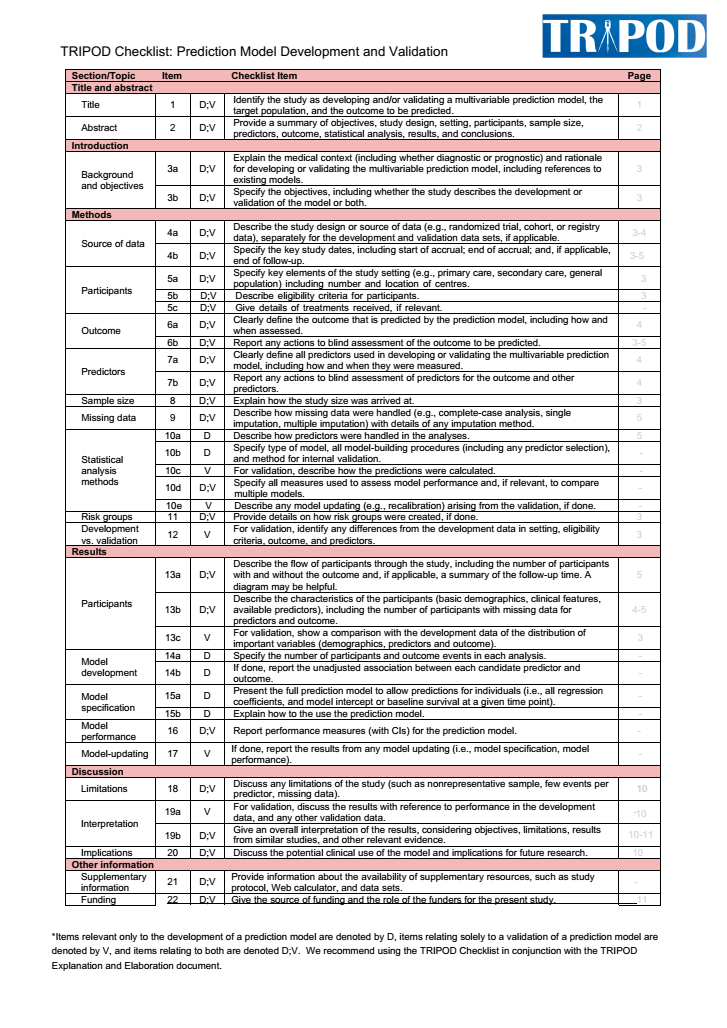

Supplement: S3 Checklist — (DOCX) [file pone.0253279.s003.docx]
