# Supplementary material for: Prognosis of amyotrophic lateral sclerosis with cognitive and behavioural changes based on a sixty-month longitudinal follow-up
Source: PLoS One. 2021 Aug 11;16(8):e0253279. doi: 10.1371/journal.pone.0253279 (PMC8357115; doi:10.1371/journal.pone.0253279)
Supplement: Attachment — Submitted filename: Revisione PLOSone - Fan.docx [file pone.0253279.s012.docx]

This is an original article about the role of cognitive impairment as a prognostic determinant in a small and heterogeneous group of ALS patient. Despite the interesting topic, the article completely lacks data to support its conclusions. First, the definition of cognitive impairment did not follow correctly the mentioned Strong criteria for ALS-FTD; also, the author used only ECAS to define cognitive categories. Second, the statistical analysis for survival is not a survival analysis (Kaplan-Meier or Cox proportional HM) but a correlation analysis, performed only on deceased patients – a significant source of bias. Third, there is no statistical comparison of the three, small and heterogeneous groups and no adjustment for other prognostic determinant, such as age, sex, bulbar vs spinal onset. The article should not be published in the current form.

Abstract:

1. The ALS-FTD is ALS plus frontotemporal dementia and not only behavioral variant, which is the most common. Please rephrase.
2. Less than half of the patients were followed-up (42 from 87).
3. Survival time is in years?

Materials and methods

4. As in the abstract, check the definition for ALS-FTD (ALS with fronto-temporal dementia) using Strong Criteria.
5. In this section, the authors did not mention any details about the neuropsychological battery used to derive cognitive classification, did they use only the ECAS? Moreover to clarify this process, I think it is better to classify patients in ALL the Strong categories contained in the Axix II of the original article (ALS-CN, ALS-ci, ALS-bi, ALS-cbi, and ALS-FTD) and then, due to the relative small number of patients included, to merge intermediate cognitive impairment into a unique category. If only ECAS was used to define cognitive status, it is very difficult to believe in this classification.
6. ECAS score published previously: citation? I think it is better to reassume patients features also in this article, not only adding the clinical information of the 3 ALS-FTD patients added. For this, Table 1 add no useful information without comparison to the other patients included.
7. If the authors said that all patients were followed up, why the only a half of patients complete the follow up? Do you mean they died? Please clarify this point.
8. Substitute “clinical death” with “death”.
9. Statistics Pearson and Spearman correlation are not survival analysis. This is the major problem of the article, use this statistical methods, combined with a small number of patients, analyzing only data from patients who reach the outcome is a huge source of bias.

Results

10. Table 2. There is no comparison among groups… for example, the disease duration is very different among groups, especially in the CN-group the survival is significantly higher than in other groups.
11. The reduced survival could be related to other clinical features, more than cognitive impairment, such as age (62 vs 55), male sex, the higher percentage of bulbar-onset patients.. without Cox analysis the authors are not able to consider all this features in adjustments.

Conclusions

12. The conclusions continue to refer only to behavioral impairment, misinterpreting the Strong definitions for ALS-FTD. Considering the multiple possible bias performed in the Results section, they should be completely rewritten.
