# Supplementary material for: Prognosis of amyotrophic lateral sclerosis with cognitive and behavioural changes based on a sixty-month longitudinal follow-up
Source: PLoS One. 2021 Aug 11;16(8):e0253279. doi: 10.1371/journal.pone.0253279 (PMC8357115; doi:10.1371/journal.pone.0253279)
Supplement: Attachment — Submitted filename: Reply to reviewer.docx [file pone.0253279.s014.docx]

**The authors revised the manuscript and responded all reviewer comments point to point. Still, I had some comment which had not been clarified. The major concerns were still the methodology and statistical analysis.**

**1. In the method section, patients with severe physical dysfunction were excluded in this study. The high rate of missing data was reasonable. However, it is essential to compare those patients with follow-up and no follow-up and to tell if the assumptions were made about verification (ignorable or missing at random–MAR) or non-ignorable or missing not at random–MNAR). The authors responsed “According to your advice, we looked back our data carefully. We didn’t contain this part of patient in the original manuscript, it might bring bias as you mentioned. ” No details about those patients was listed yet. The authors need to consider a column added to table 1 and table 2.**

Reply: Thank you very much for your constructive suggestion. According to your advice, before the results part “Analysis of ALS patients followed up to the endpoint”, we added another part to compare ALS patients who followed up to the endpoint and not. We found except for ECAS score, actually there was no difference in other clinical factors. Since the percentage of followed up to the endpoint patients was much higher in ALS-bvFTD (3/4), and ECAS score of ALS-bvFTD patients are usually lower, we get rid of 4 ALS-bvFTD patients. Then there was also no difference of ECAS score between these two groups. According to these results, we then analyze ALS followed up to the endpoint group. The added result part was listed as following:

**“***2. Comparison between followed up to the endpoint and not to the endpoint groups*

*Except for ECAS score, there was no significant difference between the ALS patients who followed up to the endpoint and not to the endpoint (Table2). The percentage of followed up to the endpoint patients was much higher in ALS-bvFTD (3/4), and ECAS score of ALS-bvFTD patients are usually lower. So when we got rid of the ALS-bvFTD patients, there was no difference of ECAS score between the two groups (t=1.55, p=0.12).*

*Table2. Comparison between followed up to the endpoint and not to the endpoint groups*

|  | *Followed up to the endpoint(n=42)* | *Not followed up to the endpoint(n=45)* | *p* |
| --- | --- | --- | --- |
| *Group* |  |  | *0.41* |
| *Pure ALS* | *18* | *20* |  |
| *ALS with cognitive and behavioural* | *31* | *24* |  |
| *ALS-bvFTD* | *3* | *1* |  |
| *Gender* |  |  | *0.82* |
| *Male* | *30* | *31* |  |
| *Female* | *12* | *14* |  |
| *Age（M±SD）* | *56.55±10.11* | *54.16±11.38* | *0.30* |
| *Education (years) (M±SD)* | *11.00±3.15* | *11.87±3.67* | *0.29* |
| *Duration of illness (months) (M±SD)* | *16.10±16.13* | *15.69±12.64* | *0.39* |
| *Onset of disease* |  |  | *0.52* |
| *Bulbar* | *9* | *10* |  |
| *Cervical* | *21* | *28* |  |
| *Thoracic* | *2* | *1* |  |
| *Lumbar* | *10* | *6* |  |
| *Diagnostic level* |  |  | *0.78* |
| *Definite* | *10* | *5* |  |
| *Probable* | *12* | *14* |  |
| *Laboratory-supported probable* | *13* | *14* |  |
| *Possible* | *7* | *12* |  |
| *Total ECAS score (M±SD)* | *80.60±18.87* | *90.09±19.30* | ***0.02**** |
| *FRS-R (M±SD)* | *40.24±4.34* | *42.18±4.73* | *0.05* |

**p<0.05*

”

**2. Since the missing data was unavoidable, the authors need to address censoring issue and modified their statistical strategy.**

Reply: Thank you very much for your advice. There was no missing data at baseline in our study. Since the study didn’t have so many follow-up factors, only FRS-R score, whether get to the endpoint. So actually, there was no missing data in our study. Thank you.

**3. In the results section, the authors stated that survival time of the ALS-bvFTD group was significantly shorter than that of the pure ALS group. However, since the distribution was likely not normal, the Wilcoxon test or other approaches could be used.**

Reply: Thank you very much for your kind advice. Yes, it was not normal distributed, and we have change into Mann-Whitney U test. The data has been changed as following: “*The survival time of the ALS-bvFTD group was significantly shorter than that of the pure ALS group (z= 2.72, p=0.002) or the ALS with cognitive and behavioural changes group (z = 2.66, p =0.008) (Fig 3)*.” Thanks again!

**4. The difference between pure ALS and cognitive impairment ALS was small. However, several baseline characteristics were needed to adjusted and log-rank test was need to be considered here.**

Reply: Thank you very much for your suggestion! We tried Log Rank test to do survival analysis in pure ALS group and ALS with cognitive and behavioural change group, there was no significance (p=0.79). We have added in the result part, as following:” *Log Rank test was used to compare the survival curve of pure ALS group and ALS with cognitive and behavioural change group, there was no significance (p=0.79).* ”

**5. The linear regression analysis between progression rate and follow-up time was performed. However，it might be nonlinear regression model and cubic or GEE model were worth be explored.**

Reply: Thank you very much for your kind suggestion. According to your advice, we have tried other models as you mentioned, like cubic, while the results were similar: It still has significance of the correlation between ECAS score and survival time ( F=3.65, p=0.02). However, ECAS score have no correlation with FRS-R score at enrolment, the FRS-R progression rate from onset to enrolment, or the progression rate from enrolment to endpoint (p > 0.05). So we add this sentence in the manuscript :” *The results were similar when using cubic and other regression models.*” Thanks again!
